# Early and Late Intrinsic Hand Muscle Reinnervation After End-to-Side AIN to Ulnar Motor Nerve Transfer

**DOI:** 10.1177/15589447241286263

**Published:** 2024-11-06

**Authors:** Eric C. Mitchell, Mehran Mansouri, Thomas Miller, Douglas Ross, Joshua Gillis

**Affiliations:** 1 Department of Surgery, Western University, London, Ontario, Canada; 2 Department of Physical Medicine and Rehabilitation, Western University, London, Ontario, Canada; 3 Division of Plastic and Reconstructive Surgery, Memorial University of Newfoundland, St. John’s, Canada

**Keywords:** nerve compression, nerve, cubital tunnel syndrome, nerve injury, research and health outcomes, rehabilitation, surgery

## Abstract

**Background:**

The “supercharge” end-to-side (SETS) anterior-interosseous-nerve (AIN) to ulnar-motor nerve transfer is used to improve intrinsic muscle recovery in cases of severe ulnar nerve compression or proximal axonotmetic injuries. Previous work has found differing intrinsic muscle recovery after this transfer. The objectives of this study were to examine the patterns of recovery in first dorsal interossei (FDI) and abductor digiti minimi (ADM) and the impact of AIN transfer to a specific fascicular location on the ulnar-motor nerve.

**Methods:**

A retrospective review of one fellowship-trained surgeon’s consecutive patients at a single center from December 2019 to September 2021 was conducted. Patients who had an AIN to ulnar-motor nerve transfer for any indication were included and were excluded if they had less than 9 months follow-up.

**Results:**

Seventeen patients were included (88% male, mean age 55 ± 14 years). At early follow-up, compound muscle action potential amplitudes for ADM and FDI did not increase. Compound muscle action potential amplitude for ADM significantly increased at late follow-up (*P* < .01). Average British Medical Research Council (BMRC) strength increased at early follow-up for FDI (*P* < .05), but not ADM. The proportion of patients with BMRC ≥ 3 increased for FDI (*P* < .01) and ADM (*P* < .05) at late follow-up. Volar-ulnar AIN insertion position did not have a clear effect on outcomes.

**Conclusions:**

The SETS AIN to ulnar-motor nerve transfer demonstrates clinical and electrophysiologic evidence of intrinsic muscle recovery and reinnervation, with differing recovery of outcomes. The role of specific fascicular targeting is still unclear and required further examination as does the mechanism behind differing intrinsic recovering.

## Introduction

Ulnar nerve dysfunction is a common clinical problem, with cubital tunnel syndrome being the second-most prevalent compression neuropathy.^1^ Debate still exists as to the ideal technique for surgical management, particularly surrounding those patients with intrinsic weakness and paralysis.^2–5^ The “supercharge” end-to-side (SETS) anterior-interosseous-nerve (AIN) to ulnar-motor nerve transfer is a technique used with the intention of improving intrinsic muscle recovery in cases of severe ulnar nerve compression and proximal axonotmetic injuries (axonal loss).^6,7^ Initial studies have shown promising clinical recovery of hand intrinsics after the addition of an AIN transfer during the management of ulnar neuropathy.^7–10^

Head et al, 2020, found that the first dorsal interosseous (FDI) muscle recovers to a greater extent than the abductor digiti minimi (ADM) after an AIN to ulnar-motor nerve transfer, despite ADM being the more proximal muscle. However, the underlying explanation for this finding is unknown.^11^ A recent cadaveric study examined the internal topography of the ulnar motor branch in the distal forearm and palm, finding a consistent arrangement of muscle fascicles moving radial to ulnar, from flexor pollicis brevis to FDI to ADM.^12^

At our institution, more anatomically directed coaptation during end-to-side AIN to ulnar-motor nerve transfers has been studied.^12^ Our hypothesis is that intentional placement of the AIN transfer to a specific anatomical fascicular location on the ulnar motor nerve may allow preferential recovery of intrinsic musculature. The objective of this study is to examine the clinical and electrodiagnostic patterns of recovery in FDI and ADM, both in terms of timing and overall extent of recovery.

## Materials and Methods

A retrospective review of one peripheral nerve and hand fellowship-trained surgeon’s consecutive patients at a single center from December 2019 to September 2021 was conducted. The AIN transfer was added for those patients with McGowan grade 3 ulnar neuropathy, demonstrating intrinsic wasting or palsy, or those patients with McGowan grade 2 and electrophysiologic evidence of muscle denervation, such as fibrillations. All patients who had an AIN to ulnar motor branch end-to-side (ETS) transfer performed for any indication were included. Patients were excluded if they had less than 9 months of follow-up. Institutional ethics review board approval was obtained for this study with a waiver of informed consent (REB# 2023-121976-74704). As an outline, Strengthening the Reporting of OBservational Studies in Epidemiology (STROBE) guidelines were used.

### Surgical Technique

**Figure 1.**
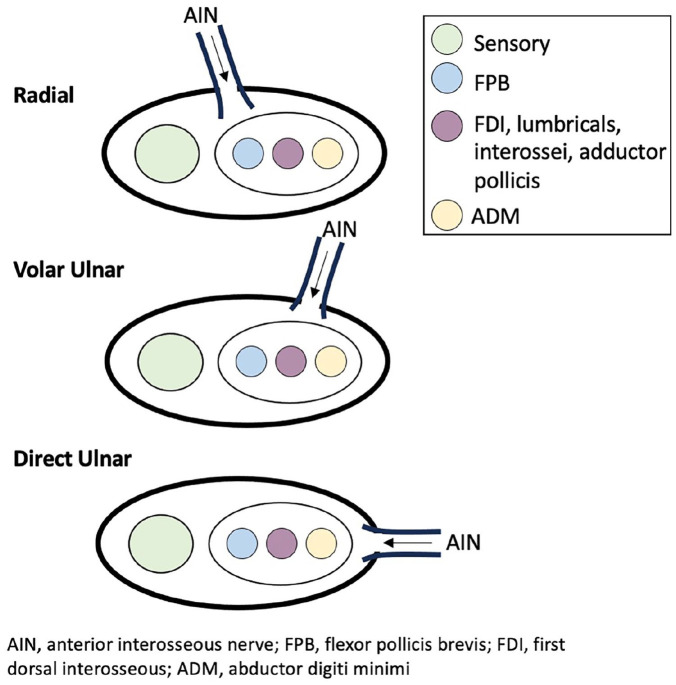
Anatomical diagram of anterior interosseous nerve coaptation location on ulnar nerve motor branch.

The typical approach for an ETS AIN to ulnar motor nerve transfer has been previously described and is used at our institution.^6^ An epineural window was made in the ulnar nerve, and the AIN was coapted end-to-side targeting a specific anatomical location on the motor branch—volar ulnar, direct ulnar, or radial (Figure 1). Direct ulnar insertion was generally used earlier in the study inclusion period as it was the initial technique performed by the senior author. With further appreciation of the ulnar nerve internal topography, the volar-ulnar insertion position was more frequently used for standard cubital tunnel compression neuropathy indications. In the volar-ulnar position, FDI fascicles may be targeted more directly at the level of the AIN transfer to attempt to preferentially improve pinch strength, as opposed to ADM fascicles.^12^ For the radial insertion, the sensory fascicles were separated from the motor fascicles to allow for AIN placement. The transfer insertion position for each patient depended on other clinical features, for example, the presence of a Martin-Gruber anastomosis with relative preservation of FDI would prompt a more direct ulnar insertion. All patients in the cohort underwent a Guyon’s canal decompression. The senior author’s preference is to do an anterior transposition at the cubital tunnel in most cases of moderate to severe neuropathy, with dynamic symptoms.^13^ Decompression and transfer surgeries were done during the same operation.

All post-operative rehabilitation was conducted by one hand therapist in a consistent fashion.^14,15^ Patients are typically seen for post-operative needle electromyography (NEE) first after 5 to 7 months and then again at 12 and 24 months from surgery. Donor-driven therapy and surface electromyography-triggered biofeedback are initiated once nascent units are seen in hand intrinsic muscles.

### Study Data Collection and Outcome Measures

Demographic, clinical, and electrodiagnostic information for all patients was obtained pre-operatively during assessment by a multi-disciplinary team consisting of three plastic surgeons and two physiatrists. Post-operative visits were divided into early (<9 months from operation) and late (>9 months from operation). Given the typical timing of post-operative visits with NEE, the earliest being 5 to 7 months, and then the subsequent visits being most often after 12 months, 9 months was chosen as a cut-off for early versus late. Primary outcome measures were ADM and FDI (British Medical Research Council (BMRC) strength, and compound muscle action potential (CMAP) amplitudes. C﻿ompound muscle action potential amplitude was measured from baseline to peak while stimulating at the level of the wrist. Post-operative BMRC strength was measured by the same multi-disciplinary team members as pre-operatively. If different raters noted different BMRC strength ratings, the lower of the two was used for this study; otherwise, the median BMRC strength rating was used.

Secondary outcomes measures were other electrodiagnostic outcomes: presence of pronation-driven nascent units (nascent motor unit potentials initiated with pronation (donor nerve) or volitional motor unit action potentials noted with donor nerve ie pronation initiated), the presence of denervation (determined by the presence of fibrillations or positive sharp waves, which reflected loss of axons) and the importance of volitional recruitment in both a pronated and supinated position of the forearm (modulation of electrodiagnostic data with pronation vs supination post-operatively on needle electromyography [EMG]). Two independent reviewers collected data from the medical record.

Sub-group analyses were completed, including an evaluation of outcomes for patients who underwent AIN insertion in the volar-ulnar position alone and a comparison of the absolute change in outcomes (BMRC strength grade and CMAP value) of patients who underwent volar-ulnar insertion and those who did not. We also examined outcomes excluding those patients that had evidence of chronic re-innervation with absent or decreased insertional activity seen in either FDI or ADM pre-operatively on NEE. Outcomes in only McGowan 3 severity patients were also assessed.

### Statistical Analysis

Data normality was assessed using the Shapiro–Wilk test. Non-parametric data was evaluated using the Wilcoxon signed rank test, the Mann–Whitney *U* test, and the McNemar change test. Correlations were evaluated using two-tailed Pearson’s coefficient. Significance was set at *P* < .05.

## Results

Twenty-four patients were eligible for this study, having undergone AIN to ulnar motor nerve transfers during the study period. Seventeen patients met inclusion criteria. Seven patients were excluded as they did not have consistent follow-up available as per the exclusion criteria. Demographic and operative details are outlined in Table 1. All patients with a compressive etiology injury type had predominant compression at the level of the elbow. Five patients had electrodiagnostic evidence of superimposed polyneuropathy. One patient had a complete high ulnar nerve transection (level of axilla) and underwent cable grafting 1 month after injury at the site of transection along with an AIN to ulnar motor nerve transfer and Guyon’s canal release. Two other patients had closed, traumatic, medial cord injuries, and were operated on at 9 and 10 months. Four patients had evidence of chronic re-innervation, seen as decreased or absent insertional activity on electrodiagnostic testing, while all other patients had evidence of acute denervation with insertional activity present. Two patients had McGowan 2 severity.

**Table 1.** Demographic, Injury and Operative Details (*n* = 17).

Characteristic	Value (%)
Sex (male)	15 (88)
Age at surgery (years)	55 ± 14
BMI	29 ± 8
Hand dominance (right)	15 (88)
Surgery on Dominant Hand (yes)	10 (59)
Diabetes (yes)	4 (24)
Hypothyroid (yes)	1 (6)
Alcohol Abuse (yes)	2 (12)
Smoking (yes)	4 (24)
Chronic ulnar nerve compression (yes)	14 (82)
Injury type	
Compressive	14 (82)
Trauma	3 (18)
Previous ipsilateral decompression/nerve-related surgery (yes)	0 (0)
Level of injury/compression	
Upper arm/plexus	3 (18)
Elbow	14 (82)
Other	0 (0)
Radiculopathy present (yes)	0 (0)
Presence of Martin-Gruber anastomosis	2 (18)
AIN insertion site	
Radial	1 (6)
Direct ulnar	3 (18)
Volar ulnar	12 (71)
Unknown	1 (6)
SETS done (yes)	17 (100)
Guyon’s canal release (yes)	17 (100)
Transposition done (yes)	14 (82)
Type of transposition	
Sub-cutaneous	1 (7)
Sub-muscular	13 (93)
Time from symptom onset to transfer surgery (months)	
Overall	24 ± 28
Acute onset	6 (range 1-9)
Chronic onset	28 (range 8-120)
Symptom onset to surgery <12-months	5 (29)
Mean follow-up (months)	17 ± 3.6

Continuous variables are presented as mean ± standard deviation. BMI, body mass index; SETS, “supercharge” end-to-side.

Age at surgery was not significantly correlated with change in BMRC value from pre-op to over 9-month follow-up for ADM (*P* = .976) or FDI (*P* = .751) or the change in CMAP for ADM (*P* = .849) or FDI (*P* = .374). Time to surgery from injury/compression onset was similarly not significantly correlated with these outcomes. There were no significant differences in the change in BMRC or CMAP values when comparing those with a traumatic etiology, compared to those without.

Average months post-op at early follow-up was 6.7 ± 0.9 months (range 4.9-8.1) and late follow-up was 17.3 ± 3.6 (range 11.7-23.4). There was no difference in the length of follow-up based on insertion position. All patients had normal pronator quadratus function on pre-operative NEE.

The pre-operative and post-operative motor amplitudes and BMRC grades for FDI and ADM can be seen in Tables 2, 3, and 4.

**Table 2.** Pre-Operative and Post-Operative Intrinsic Muscle Motor Amplitude.

Muscle		Post-Op CMAP		Post-Op CMAP	
	Pre-Op CMAP	>9-Month Follow-Up Visit	*P* *	>9-Month Follow-Up Visit	*P* *
**ADM**					
No.	17	11	.271	15	.006
Median (range)	1.3 (0-5.1)	1.2 (0-4.3)		2.3 (0-7.6)	
Mean ± SD	1.6 (1.7)	1.5 (1.5)		3.4 (2.3)	
**FDI**					
No.	16	7	1.000	9	.112
Median (range)	0.1 (0-5.9)	1.4 (0-4.0)		1.0 (0-4.2)	
Mean ± SD	1.2 (1.8)	1.2 (1.4)		1.6 (1.6)	

ADM, abductor digiti minimi; FDI, first dorsal interosseous; CMAP, compound muscle action potential.

* Wilcoxon Signed Rank Test.

**Table 3.** Pre-Operative and Post-Operative Mean Intrinsic Muscle BMRC Grade.

Muscle		Post-Op BMRC		Post-Op BMRC	
	Pre-Op BMRC Grade	<9-month follow-up visit	*P* *	>9-month follow-up visit	*P* *
**ADM**					
No.	15	12	.064	16	.003
Median (range)	1.0 (0-3)	2.0 (0-4)		3.5 (1-4)	
Mean ± SD	1.5 (1.2)	2.3 (1.4)		3.1 (1.1)	
**FDI**					
No.	17	12	.040	17	.002
Median (range)	1.0 (0-4)	2.0 (0-4)		3.0 (1-4)	
Mean ± SD	1.4 (1.2)	2.2 (1.3)		2.8 (1.1)	

ADM, abductor digiti minimi; FDI, first dorsal interosseous; BMRC, British Medical Research Council.

* Wilcoxon Signed Rank Test.

**Table 4.** Pre-Operative and Post-Operative Intrinsic Muscle Functional BMRC Grade.

Muscle		Post-Op BMRC Grade		Post-Op BMRC Grade	
	Pre-Op BMRC Grade	<9-Month Follow-Up Visit	*P* *	>9-Month Follow-Up Visit	*P* *
**ADM**					
BMRC≥ 3 (%)	4 (27)	5 (42)	.375	12 (75)	.016
BMRC < 3 (%)	11 (73)	7 (58)		4 (25)	
**FDI**					
BMRC≥ 3 (%)	3 (18)	5 (42)	.625	11 (65)	.008
BMRC < 3 (%)	14 (82)	7 (58)		6 (35)	

ADM, abductor digiti minimi; FDI, first dorsal interosseous; BMRC, British Medical Research Council.

* McNemar Test.

At early follow-up there were no significant differences in CMAP amplitudes for either ADM (*P* = .271) or FDI (*P* = 1.000). At late follow-up, CMAP amplitude for ADM was significantly increased from pre-op (*P* < .05), while the amplitude of FDI did not significantly increase.

Mean BMRC significantly increased from pre-op to early follow-up post-op for FDI (*P* < .05), but not ADM (*P* = .064). Mean BMRC significantly increased from pre-op to late follow-up for both ADM (*P* < .01) and FDI (*P* < .01). The proportion of patients with functional ADM strength (BMRC≥3) did not significant increase from pre-op to post-op at early follow-up (*P* = .375) but did increase at late follow-up (*P* < .05). The proportion of patients with functional FDI strength (BMRC ≥ 3) did not increase from pre-op to early follow-up (*P* = .625) but did increase significantly from pre-op to late follow-up (*P* < .01).

The mean number of month post-operatively to first nascent potential seen on electrodiagnostic testing for ADM was 8.7 ± 4.0 months and 10.7 ± 4.0 months for FDI.

89% of patients had evidence of FDI denervation pre-operatively, and 67% had ADM denervation. One patient did not have denervation of either FDI or ADM pre-operatively, but had a BMRC of 2 and 1 for FDI and ADM, respectively. Of the patients who had EMG done of ADM and FDI, 33% (*n* = 4) and 18% (*n* = 3) of patients demonstrated improved recruitment with donor nerve firing (pronation vs supination), respectively, at their first follow-up visit.

Tables 5 and 6 show the outcomes of patients who had AIN insertion in the volar-ulnar position (*n* = 12). The proportion of patients with functional ADM strength (BMRC ≥ 3) significantly increased at late follow-up (<.05), but not for FDI (*P* = .063). CMAP significantly increased at late follow-up for ADM (*P* = .037), but not for FDI (*P* = .176).

**Table 5.** Volar Ulnar Subgroup - Pre-Operative and Post-Operative Mean Intrinsic Muscle BMRC Grade.

Muscle		Post-Op BMRC Grade		Post-Op BMRC Grade	
	Pre-Op BMRC Grade	<9-Month Follow-Up Visit	*P* *	>9-Month Follow-Up Visit	*P* *
**ADM**					
No.	11	10	.064	11	.017
Mean ± SD	1.6 (1.2)	2.5 (1.3)		3.2 (1.0)	
**FDI**					
No.	12	10	.04	12	.007
Mean ± SD	1.2 (1.1)	2.1 (1.2)		2.7 (1.1)	

ADM, abductor digiti minimi; FDI, first dorsal interosseous; BMRC, British Medical Research Council.

* Wilcoxon Signed Rank Test.

**Table 6.** Volar Ulnar Subgroup - Pre-Operative and Post-Operative Intrinsic Muscle Functional BMRC Grade.

Muscle		Post-Op BMRC Grade		Post-Op BMRC Grade	
	Pre-Op BMRC Grade	<9-Month Follow-Up Visit	*P* *	>9-Month Follow-Up Visit	*P* *
**ADM**					
BMRC≥ 3 (%)	3 (27)	4 (40)	.375	9 (82)	.031
BMRC < 3 (%)	8 (73)	6 (60)		2 (18)	
**FDI**					
BMRC≥ 3 (%)	2 (17)	4 (40)	.625	7 (58)	.063
BMRC < 3 (%)	10 (83)	6 (60)		5 (42)	

ADM, abductor digiti minimi; FDI, first dorsal interosseous; BMRC, British Medical Research Council.

* McNemar Test.

Supplemental Tables S1 and S2 show the comparisons between patients who had a volar-ulnar insertion of the AIN compared to those not inserted volar-ulnar (“other” group—direct ulnar, radial or unknown). Only patients who had both pre-op and post-op values were included. There were no significant differences seen with any comparison. We were unable to compare the change in FDI CMAP due to insufficient sample size.

When patients with absent or decreased insertional activity with or without sparse denervation potentials and or CRDs were excluded, there were no significant differences in CMAP amplitudes for ADM (*P* = .104) or FDI (.593) at early follow-up, while ADM CMAP significantly increased at late follow-up (*P* < .05). Mean BRMC significantly increased from pre-op to late follow-up for ADM (*P* < .05) and FDI (*P* < .05). When patients with McGowan 2 grade changes (*n* = 2) were excluded from the analysis the results remained the same in terms of significant and non-significant findings.

## Discussion

In this cohort of 17 patients, we demonstrate clinical and electrophysiologic evidence of hand intrinsic muscle reinnervation after supercharge end-to-site AIN to ulnar-motor nerve transfer. The proportion of study patients achieving BMRC grade 3 or greater strength in both ADM and FDI is similar to previously described results.^7,8,11,16^ Head et al found 71% of patients achieved BMRC grade 3 or greater for FDI and 65% for ADM at mean follow-up of 16.7 months, compared to our findings of 65% and 75%, respectively.^11^

The timing of intrinsic recovery after this nerve transfer has been examined in previous studies.^7,8^ Davidge et al looked at the rate and timing of intrinsic recovery post-operative from AIN to ulnar-motor nerve transfer.^7^ They found 49% of patients demonstrated gradual recovery of FDI muscle function between 1 and 3 months and 6 and 12 months post-operatively. Sixteen percent of patients had evidence of rapid intrinsic recovery (by 3-month follow-up), considered to be secondary to reversal of conduction block, whereas axonal regeneration occurs at a slower pace of 1 mm per day.^7^ In our study, no patients had post-operative electrodiagnostic testing done before 5 months, as such we were not able to examine the frequency of rapid recovery. Our results do indicate some recovery of FDI by less than 9-month post-operative with significant improvement in mean BMRC score, which would not be possible through axon regeneration from the level of the cubital tunnel given the severity of pre-operative changes, but could potentially result from collateral sprouting of remaining ulnar-motor nerve axons. Some contribution of the AIN nerve transfer is suggested by the findings of ADM and FDI electrodiagnostic data with pronation versus supination at first follow-up and the presence of nascent motor unit potentials initiated with pronation seen in ADM and FDI at a mean of 9 and 11 months post-operatively, respectively. Doherty et al 2020 reported a mean time of 9 ± 5 months to see nascent units in ADM, FDI or fourth DI.^16^ In this study we were unable to separate the amount of intrinsic recovery related to Guyon’s canal decompression or proximal transposition as opposed to the nerve transfer, given all patients had a Guyon’s decompression and most patients had a transposition.

Previous work found differential recovery of hand intrinsic musculature after AIN to ulnar-motor nerve transfer, with FDI achieving significantly greater post-operative strength compared to ADM at a mean follow-up of 16.7 months, while both seeing a significant increase in CMAP amplitude.^11^ This is a somewhat counter-intuitive finding as recovery of FDI should be less than that of ADM, at least in the initial stages, as the nerve must regenerate over a longer distance. Re-innervation from the nerve transfer to ADM is around the half-way point to re-innervation of FDI, based on recovery at 1 mm per day. Two hypotheses were provided for this finding, one being that the anatomical location of the epineural window for end-to-side coaptation could lead to preferential targeting and therefore recovery of one distal motor fascicle more than another. The other suggestion is post-operative therapy and rehabilitation could favor recovery of one muscle more than another.^11^ In our study, we found some differential intrinsic recovery, with FDI achieving a significant increase in BMRC at early follow-up, while ADM did not; a counterintuitive finding similar to the findings reported by Head et al^11^ Earlier significant improvement of ADM would fit with recovery of intrinsic muscles based purely on distance to muscle motor endplates, but this was not seen.

Three of our cohort patients did not achieve an BMRC ≥ 3 in at least one intrinsic muscle which has been considered a “failure” in a previous study.^8^ These patients were followed for 13, 18, and 22 months post-operatively, which should have been adequate to record intrinsic recovery. Older age at surgery has been shown to be a negative predictor of nerve transfer failure.^17,18^ The ages of these three patients at time of surgery were 51, 54, and 61, which is in line with the mean age in the cohort of 55. Only one patient had absent CMAPs in ADM and FDI pre-operatively. All three had normal or high insertional activity in FDI or ADM pre-operatively. We did not identify other specific clinical variables that could explain why the transfer was unsuccessful in these patients.

The intentional targeting of specific fascicles during nerve coaptation has been applied in both upper and lower extremity nerve transfers, but not as employed in this study’s surgical technique by altering the location of AIN insertion on the ulnar motor nerve.^19–21^ Most studies describing their specific technique for the AIN to ulnar-motor nerve transfer target the motor component of the ulnar nerve in general through an intra-fascicular dissection.^6,9,16^ In a recent study of Canadian plastic surgeons, most surgeons believed specific AIN placement targeting the motor fascicles makes a difference in outcomes, with varying preferences and consistency in the exact location.^22^ A recent investigation of the internal topography of the ulnar nerve motor branch found anatomical consistency of the fascicles, which was maintained as the branch continued into the palm, with ADM fascicles occupying the ulnar-most position and FDI fascicles being immediately radial to that.^12^ The senior author of this paper, who performed all operations, has taken to specifically targeting coaptations to direct locations on the ulnar nerve motor branch. In 71% of cases, the volar-ulnar position was targeted, which, based on the internal topography, was hypothesized to possible target FDI more intentionally.^12^ In our study, FDI had a significant increase in mean BMRC at early follow-up, while ADM did not, however both achieved a significant increase at late follow-up. This early follow-up finding was also seen when examining the volar-ulnar insertion patients alone. In theory, donor AIN axons placed nearer to a specific fascicle may produce a more favorable recovery of that muscle group. The potential mechanism of this is unclear, whether it be through more directed collateral sprouting, a larger positive neurotrophic “babysitting” effect on the recovery ulnar-motor nerve, or that the number of axons in the AIN donor is not sufficient to reinnervate all ulnar-innervated hand musculature, or a combination.^16,23,24^ A larger sample size of patients and further neurophysiological investigations would be needed to help answer this question. In this study we looked to compare outcomes between the volar-ulnar group and those patients with an alternate insertion position, however the small sample limited this analysis. With the overall goal of optimizing intrinsic recovery, FDI in particular, another surgical technique that has been suggested is the “super turbocharge end-to-side transfer” which combines an AIN to ulnar motor nerve transfer with a distal ADM branch transfer to the more radial side of the deep motor nerve.^25^ This similarly takes advantage of our understanding of the ulnar nerve topography in an attempt to direct recovering axons preferentially toward FDI.

This study has a number of limitations, one being the small sample size and heterogeneity of the study population, but all patients have moderate to severe ulnar neuropathy with axonal loss. Both acute (trauma, three patients) and chronic cases were included in this cohort, as well as patients with various injury etiologies. Larger sample size studies are needed to conduct more advanced sub-group analyses. Second, the reporting of additional clinical outcomes like grip, tripod, and key pinch strength as well as abduction-adduction drawings was inconsistent, therefore unable to be used in our analysis to correlate to intrinsic recovery. It is well documented both in clinical practice and throughout the literature that relying on BMRC grade alone as a clinical outcome has its shortcomings as the validity of BMRC scoring has been fraught with large coefficients of variation between observers in the literature.^26,27^ We took the lesser of the BMRC grading if scores were different among the physicians in the multi-disciplinary clinic to try to account for some of this inherent bias. The inconsistency in reporting outcomes in this population has been previously highlighted and given the retrospective nature of this paper strict adherence to donor-driven post-operative therapy was unable to evaluated. This is considered a crucial part of optimizing outcomes after this nerve transfer and the protocol used at our center is reviewed with every patient by our hand therapist at each clinic visit and has been previously described.^14,15^ Third, as in any retrospective study, recall bias and variable documentation of primary and secondary outcomes exists. Another potential confounding factor is surgeon expertise. The operations done with a volar-ulnar insertion were typically done later in the study inclusion period compared to the other positions, raising the possibility that outcomes may be better as more experience is gained. Ultimately, we did not see any significant differences based on insertion position. Finally, nerve recovery is known to continue well into the post-operative period and our mean follow-up of 17 months is not sufficient to gauge or ascertain final level of recovery. Given the availability of post-operative NEE data we were unable to evaluate intrinsic recovery at more than two post-operative time-points, which could potentially reveal evidence of further differential recovery. A prospective randomized trial comparing placement of the AIN at different sites of the ulnar motor fascicles in ulnar nerve entrapment injuries with axonal loss would help to further elucidate whether this changes the overall reinnervation pattern of the intrinsic musculature and ultimately impacts functional outcome. The current study suggests it may be an additional and important factor in SETS transfers in this context.

## Conclusions

We conclude that the ETS AIN to ulnar-motor branch transfer demonstrates clinical and electrophysiologic evidence of intrinsic muscle recovery and reinnervation at both early and late follow-up, with some differential recovery. The role of specific fascicular targeting is still unclear and requires further investigation.

## Supplemental Material

sj-docx-1-han-10.1177_15589447241286263 – Supplemental material for Early and Late Intrinsic Hand Muscle Reinnervation After End-to-Side AIN to Ulnar Motor Nerve Transfer Supplemental material, sj-docx-1-han-10.1177_15589447241286263 for Early and Late Intrinsic Hand Muscle Reinnervation After End-to-Side AIN to Ulnar Motor Nerve Transfer by Eric C. Mitchell, Mehran Mansouri, Thomas Miller, Douglas Ross and Joshua Gillis in HAND

sj-docx-2-han-10.1177_15589447241286263 – Supplemental material for Early and Late Intrinsic Hand Muscle Reinnervation After End-to-Side AIN to Ulnar Motor Nerve Transfer Supplemental material, sj-docx-2-han-10.1177_15589447241286263 for Early and Late Intrinsic Hand Muscle Reinnervation After End-to-Side AIN to Ulnar Motor Nerve Transfer by Eric C. Mitchell, Mehran Mansouri, Thomas Miller, Douglas Ross and Joshua Gillis in HAND

## References

[1] An TW Evanoff BA Boyer MI , et al The prevalence of cubital tunnel syndrome: a cross-sectional study in a U.S. Metropolitan cohort. J Bone Joint Surg Am. 2017;99(5):408–416. doi:10.2106/JBJS.15.01162. 28244912 PMC5324036

[2] Adkinson JM Zhong L Aliu O , et al Surgical treatment of cubital tunnel syndrome: trends and the influence of patient and surgeon characteristics. J Hand Surg. 2015;40(9):1824–1831. doi:10.1016/j.jhsa.2015.05.009 PMC4819985 26142079

[3] Gervasio O Gambardella G Zaccone C , et al Simple decompression versus anterior submuscular transposition of the ulnar nerve in severe cubital tunnel syndrome: a prospective randomized study. Neurosurgery. 2005;56(1):108. doi:10.1227/01.NEU.0000145854.38234.81 15617592

[4] Zlowodzki M Chan S Bhandari M , et al Anterior transposition compared with simple decompression for treatment of cubital tunnel syndrome: a meta-analysis of randomized controlled trials. JBJS. 2007;89(12):2591. 10.2106/JBJS.G.00183 18056489

[5] Izadpanah A Gibbs C Spinner RJ , et al Comparison of in situ versus subcutaneous versus submuscular transpositions in the management of McGowan stage III cubital tunnel syndrome. Hand N Y N. 2021;16(1):45–49. doi:10.1177/1558944719831387 PMC7818036 30907136

[6] Barbour J Yee A Kahn LC , et al Supercharged end-to-side anterior interosseous to ulnar motor nerve transfer for intrinsic musculature reinnervation. J Hand Surg. 2012;37(10):2150–2159. doi:10.1016/j.jhsa.2012.07.022 23021177

[7] Davidge KM Mackinnon SE. The supercharge end-to-side anterior interosseous to ulnar motor nerve transfer for restoring intrinsic function: clinical experience. J Hand Surg. 2013;38:e21–e22. 10.1097/PRS.0000000000001514 26313839

[8] Dengler J Dolen U Patterson JMM , et al Supercharge end-to-side anterior interosseous-to-ulnar motor nerve transfer restores intrinsic function in cubital tunnel syndrome. Plast Reconstr Surg. 2020;146(4):808–818. 32590517 10.1097/PRS.0000000000007167

[9] McLeod GJ Peters BR Quaife T , et al Anterior interosseous-to-ulnar motor nerve transfers: a single center’s experience in restoring intrinsic hand function. HAND. 2022;17(4):609–614. 32696669 10.1177/1558944720928482 PMC9274878

[10] Baltzer H Woo A Oh C , et al Comparison of ulnar intrinsic function following supercharge end-to-side anterior interosseous-to-ulnar motor nerve transfer: a matched cohort study of proximal ulnar nerve injury patients. Plast Reconstr Surg. 2016;138:1264–1272. 27879594 10.1097/PRS.0000000000002747

[11] Head LK Zhang ZZ Hicks K , et al Evaluation of intrinsic hand musculature reinnervation following supercharge end-to-side anterior interosseous-to-ulnar motor nerve transfer. Plast Reconstr Surg. 2020;146(1):128–132. 32590654 10.1097/PRS.0000000000006903

[12] Chambers SB Wu KY Smith C , et al Interfascicular anatomy of the motor branch of the ulnar nerve: a cadaveric study. J Hand Surg. 2023;48(309):e1–309.e6. 10.1016/j.jhsa.2021.10.012 34949481

[13] Dellon AL Coert JH. Results of the musculofascial lengthening technique for submuscular transposition of the ulnar nerve at the elbow. JBJS. 2004;86(1):169–179. 10.2106/00004623-200409001-00007 15466757

[14] Tsang P Larocerie-Salgado J MacDermid JC , et al Postoperative management and rehabilitation after the supercharged end-to-side anterior interosseous nerve to ulnar motor nerve transfer: a report of 3 cases. J Hand Ther. 2021;34(3):469–478. 32571598 10.1016/j.jht.2020.03.021

[15] Larocerie-Salgado J Chinchalkar S Ross DC , et al Rehabilitation following nerve transfer surgery. Tech Hand Up Extrem Surg. 2022;26(2):71–77. 34619740 10.1097/BTH.0000000000000359

[16] Doherty CD Miller TA Larocerie-Salgado J , et al Reverse end-to-side anterior interosseous nerve-to-ulnar motor transfer for severe ulnar neuropathy. Plast Reconstr Surg. 2020;146(3):306e–313e. 10.1097/PRS.0000000000007059 32842108

[17] Verdú E Ceballos D Vilches JJ , et al Influence of aging on peripheral nerve function and regeneration. J Peripher Nerv Syst JPNS. 2000;5(4):191–208. 11151980 10.1046/j.1529-8027.2000.00026.x

[18] Vaughan DW. Effects of advancing age on peripheral nerve regeneration. J Comp Neurol. 1992;323(2):219–237. 1401257 10.1002/cne.903230207

[19] Wells ME Gonzalez GA Childs BR , et al Radial to axillary nerve transfer outcomes in shoulder abduction: a systematic review. Plast Reconstr Surg – Glob Open. 2020;8(9):e3096. 10.1097/GOX.0000000000003096 PMC7544396 33133948

[20] Giuffre JL Bishop AT Spinner RJ , et al Partial tibial nerve transfer to the tibialis anterior motor branch to treat peroneal nerve injury after knee trauma. Clin Orthop. 2012;470:779–790. 21626085 10.1007/s11999-011-1924-9 PMC3270157

[21] Tung TH Mackinnon SE. Nerve transfers: indications, techniques, and outcomes. J Hand Surg. 2010;35(2):332–341. 10.1016/j.jhsa.2009.12.002 20141906

[22] Chambers SB Wu KY Ross DC Gillis JA. Anterior interosseus to ulnar motor nerve transfers: a Canadian perspective. HAND. 2023;15589447231174482. 10.1177/15589447231174482 PMC11483773 37341212

[23] Konofaos P Bassilios Habre S Wallace RD. End-to-side nerve repair: current concepts and future perspectives. Ann Plast Surg. 2018;81(6):736–740. 30362965 10.1097/SAP.0000000000001663

[24] Schenck TL Stewart J Lin S , et al Anatomical and histomorphometric observations on the transfer of the anterior interosseous nerve to the deep branch of the ulnar nerve. J Hand Surg Eur. 2015;40(6):591–596. 10.1177/1753193414551909 25261412

[25] Peters BR Jacobson L Pripotnev S , et al Abductor digiti minimi and anterior interosseous to ulnar motor nerve transfer: the super turbocharge end-to-side transfer. Plast Reconstr Surg. 2023;151(4):815–820. 36729855 10.1097/PRS.0000000000010003

[26] Shahgholi L Bengtson KA Bishop AT , et al A comparison of manual and quantitative elbow strength testing. Am J Phys Med Rehabil. 2012;91(10):856–862. 22854900 10.1097/PHM.0b013e31825f14f9

[27] Paternostro-Sluga T Grim-Stieger M Posch M , et al Reliability and validity of the Medical Research Council (MRC) scale and a modified scale for testing muscle strength in patients with radial palsy. J Rehabil Med. 2008;40(8):665–671. 19020701 10.2340/16501977-0235

