# Supplementary material for: Early and Late Intrinsic Hand Muscle Reinnervation After End-to-Side AIN to Ulnar Motor Nerve Transfer
Source: Hand (N Y). 2024 Nov 6;20(8):1207–15. doi: 10.1177/15589447241286263 (PMC11559903; doi:10.1177/15589447241286263)
Supplement: sj-docx-1-han-10.1177_15589447241286263 – Supplemental material for Early and Late Intrinsic Hand Muscle Reinnervation After End-to-Side AIN to Ulnar Motor Nerve Transfer [file sj-docx-1-han-10.1177_15589447241286263.docx]

|  | |  |  | |  |
| --- | --- | --- | --- | --- | --- |
| **ADM MRC Pre-Op to <9 Months** | Volar-Ulnar | | Other | p | |
| No. | 9 | | 2 | 0.34 | |
| Median (range) | 1 (-1-3) | | 0 (0-0) |  | |
| Mean ± SD | 1 (1.4) | | 0 (0) |  | |
|  |  | |  |  | |
| **ADM MRC Pre-Op to >9 Months** | Volar-Ulnar | | Other | p | |
| No. | 11 | | 4 | 1.00 | |
| Median (range) | 2 (0-4) | | 1 (1-3) |  | |
| Mean ± SD | 1.6 (1.5) | | 1.5 (1.0) |  | |

Supplemental Table S1. Comparison of Change in Intrinsic Muscle BMRC for Volar-Ulnar Insertion Position and Other Insertion Positions

| **FDI MRC Pre-Op to <9 Months** | Volar-Ulnar | Other | p |
| --- | --- | --- | --- |
| No. | 10 | 2 | 0.26 |
| Median (range) | 1 (-1-3) | 0 (0-0) |  |
| Mean ± SD | 1 (1.2) | 0 (0) |  |
|  |  |  |  |
| **FDI MRC Pre-Op to >9 Months** | Volar-Ulnar | Other | p |
| No. | 12 | 5 | 0.65 |
| Median (range) | 1.5 (0-3) | 1 (0-3) |  |
| Mean ± SD | 1.5 (1.2) | 1.2 (1.3) |  |

^ADM, abductor digiti minimi; FDI, first dorsal interosseous; MRC, Medical Research Council^

^Other – insertion location other than volar-ulnar (i.e. direct ulnar, radial, unknown)^

^*Mann-Whitney U Test^
