# Supplementary material for: Early and Late Intrinsic Hand Muscle Reinnervation After End-to-Side AIN to Ulnar Motor Nerve Transfer
Source: Hand (N Y). 2024 Nov 6;20(8):1207–15. doi: 10.1177/15589447241286263 (PMC11559903; doi:10.1177/15589447241286263)
Supplement: sj-docx-2-han-10.1177_15589447241286263 – Supplemental material for Early and Late Intrinsic Hand Muscle Reinnervation After End-to-Side AIN to Ulnar Motor Nerve Transfer [file sj-docx-2-han-10.1177_15589447241286263.docx]

Supplemental Table S2. Comparison of Change in Intrinsic Muscle Motor Amplitude for Volar-Ulnar Insertion Position and Other Insertion Positions

|  | | | | | | | | |
| --- | --- | --- | --- | --- | --- | --- | --- | --- |
|  |  | |  | |  | |  |  |
| **ADM CMAP Pre-Op to <9 Months** | | Volar-Ulnar | | Other | | p | |  |
| No. | | 9 | | 2 | | 0.91 | |  |
| Median (range) | | 0.0 (-1.7-1.4) | | -0.1 (-0.2-0) | |  | |  |
| Mean ± SD | | -0.3 (0.9) | | -0.1 (0.1) | |  | |  |
|  | |  | |  | |  | |  |
| **ADM CMAP Pre-Op to >9 Months** | | Volar-Ulnar | | Other | | p | |  |
| No. | | 10 | | 5 | | 1.00 | |  |
| Median (range) | | 1.2 (-1.7-7.4) | | 1.6 (-0.4-4.4) | |  | |  |
| Mean ± SD | | 2.1 (2.8) | | 1.8 (1.8) | |  | |  |

|  | |  | |  | | |  |
| --- | --- | --- | --- | --- | --- | --- | --- |
| **FDI CMAP Pre-Op to <9 Months** | Volar-Ulnar | | Other | | p |  |  |
| No. | 6 | | 0 | | - |  |  |
| Median (range) | -0.1 (-0.4-1.5) | | - | |  |  |  |
| Mean ± SD | 0.2 (0.7) | | - | |  |  |  |
|  |  | |  | |  |  |  |
| **FDI CMAP Pre-Op to >9 Months** | Volar-Ulnar | | Other | | p |  |  |
| No. | 8 | | 1 | | - |  |  |
| Median (range) | 0.1 (-0.3-3.2) | | 0.3 | |  |  |  |
| Mean ± SD | 0.7 (1.3) | | 0.3 | |  |  |  |

^ADM, abductor digiti minimi; FDI, first dorsal interosseous; CMAP, compound muscle action potential^

^Other – insertion location other than volar-ulnar (i.e. direct ulnar, radial, unknown)^

^*Mann-Whitney U Test^
